# 428. Severe Acute Respiratory Syndrome Coronavirus 2 Household Transmission during the Omicron Era in Massachusetts: A Prospective, Case-Ascertained Study using Genomic Epidemiology

**DOI:** 10.1093/ofid/ofae631.142

**Published:** 2025-01-29

**Authors:** Jaspreet Banga, Taylor M Brock-Fisher, Brittany A Petros, Ariana T Leonelli, Katelyn S Messer, Audrey Nathanson, Viola Appiah-Danquah, Siang Dim, Maura Crowther, Kannon Lee, Katherine C DeRuff, Bronwyn MacInnis, Michael Springer, Pardis C Sabeti, Kathryn E Stephenson

**Affiliations:** Beth Israel Deaconess Medical Center, Boston, Massachusetts; Harvard University, Cambridge, Massachusetts; Broad Institute of MIT & Harvard, Cambridge, Massachusetts; Harvard University, Cambridge, Massachusetts; Broad Institute of Harvard and MIT, Boston, MA; BIDMC, Boston, Massachusetts; Beth Israel Deaconess Medical Center, Boston, Massachusetts; Beth Israel Deaconess Medical Center, Boston, Massachusetts; Beth Israel Deaconess Medical Center, Boston, Massachusetts; Harvard University, Cambridge, Massachusetts; Broad Institute, Cambridge, Massachusetts; Broad Institute, Cambridge, Massachusetts; Harvard Medical School, Boston, Massachusetts; Harvard TH Chan School of Public Health, Cambridge, MA; Harvard Medical School, Boston, Massachusetts

## Abstract

**Background:**

Households are a major setting for SARS-CoV-2 infections, but there remains a lack of knowledge regarding the dynamics of viral transmission, particularly in the setting of pre-existing SARS-CoV-2 immunity and evolving variants.

**Table 1.**
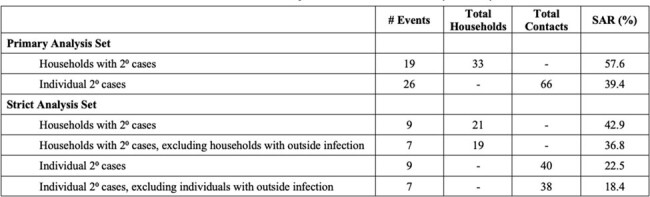
SARS-CoV-2 Secondary Attack Rates (SAR)

**Methods:**

We conducted a prospective, case-ascertained household transmission study in the greater Boston area in March-July 2022. Anterior nasal swabs, along with clinical and demographic data, were collected for 14 days. Nasal swabs were tested for SARS-CoV-2 by PCR. Whole genome sequencing was performed on high-titer samples.

**Figure 1.**
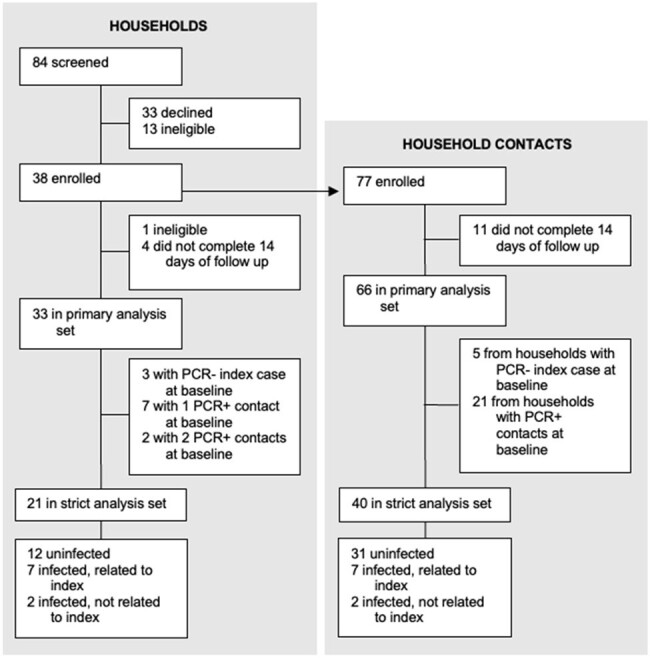
Trial profile

**Results:**

We enrolled 33 households in a primary analysis set, with a median age of participants of 25 years old (range 2-66), 98% of whom had received at least 2 doses of a COVID-19 vaccine. 58% of households had a secondary case during follow up and the secondary attack rate (SAR) for contacts was 39%. We further examined a strict analysis set of 21 households that had only 1 PCR+ case at baseline, finding an SAR of 22.5%. Genomic epidemiology further determined that there were multiple sources of infection for household contacts, including the index case and outside introductions. When limiting estimates to only highly probable transmissions given epidemiologic and genomic data, the SAR was 18.4%.

**Figure 2.**
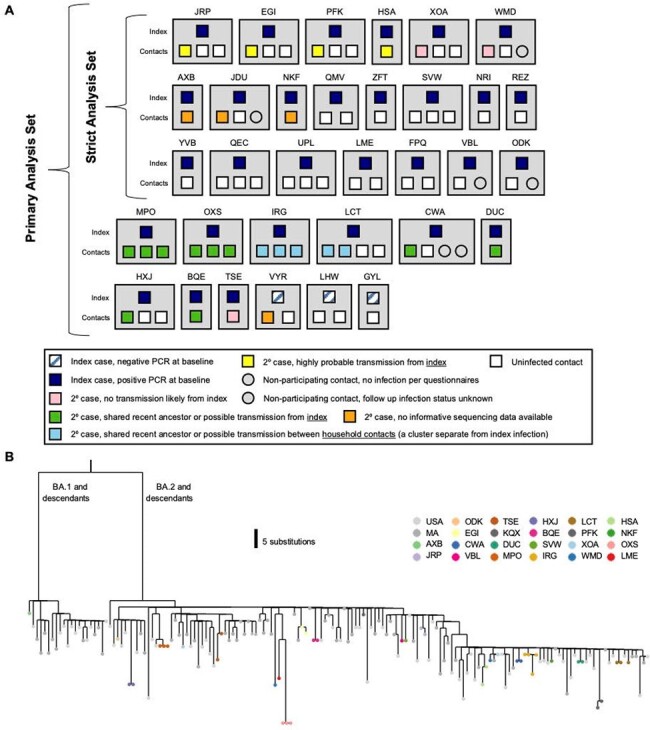
Household diagram (A) Individual households are labeled with a 3-letter identifier and represented with grey boxes. Households in both the primary and strict analysis sets are shown. Each member of the household is shown within the grey box, with their outcome color-coded as described in the legend. (B) Phylogenetic map of positive samples.

**Conclusion:**

Household contacts of a person newly diagnosed with COVID-19 are at high risk for SARS-CoV-2 infection in the following 2 weeks. This is, however, not only due to infection from the household index case, but also because the presence of an infected household member implies increased SARS-CoV-2 community transmission.

**Figure 3.**
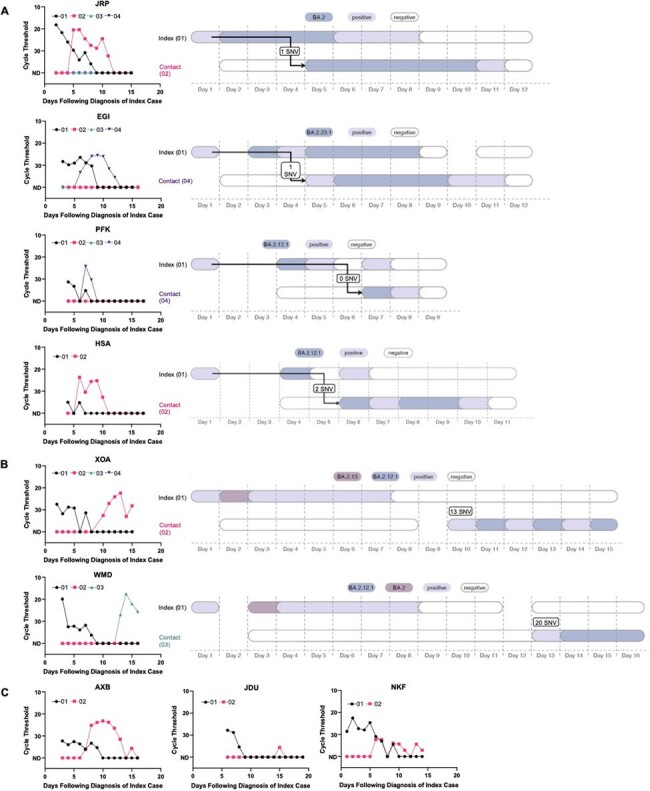
Viral load curves and transmission plots by household in the strict analysis set (A) Households where transmission was determined to be highly probable are shown. (B) Households where transmission was determined to be unlikely are shown. (C) Households that did not yield sufficient sequencing data are shown.

**Disclosures:**

**Michael Springer, Ph. D.**, Nexus laboratories: Advisor/Consultant|Nexus laboratories: Stocks/Bonds (Private Company)|Rhinostics: Board Member|Rhinostics: Ownership Interest|Rhinostics: Stocks/Bonds (Private Company) **Pardis C. Sabeti, MD, PhD**, Danaher Corporation: Board Member|Danaher Corporation: Stocks/Bonds (Public Company)|Delve Bio: Co-founder|Delve Bio: Ownership Interest|NextGenJane: former SAB member, investor|NextGenJane: Ownership Interest|Polaris Genomics: investor|Polaris Genomics: Ownership Interest|Sherlock Biosciences: Patents for CRISPR-based and microfluidic based diagnostics licensed|Sherlock Biosciences: Co-founder|Sherlock Biosciences: Ownership Interest

